# “It Happened When I Was Connecting to the Community…”: Multiple Pathways to Migrant (Non)Belonging in a New Destination Setting

**DOI:** 10.3390/ijerph20032172

**Published:** 2023-01-25

**Authors:** Claudia Soto Saavedra, Jane Lilly Lopez, Stacey A. Shaw, Benjamin G. Gibbs

**Affiliations:** Departments of Sociology and Social Work, Brigham Young University, Provo, UT 84602, USA

**Keywords:** migration, belonging, migrant status, wellbeing

## Abstract

Migrants’ sense of belonging in their country and community of residence has direct effects on their health and wellbeing. A diverse set of case studies suggest that legal immigration status plays a primary role in shaping migrants’ opportunities for and experiences of belonging. Few of these studies, though, have examined belonging for migrants with varied legal immigration statuses living in the same receiving context, limiting our understanding of if and how migrant status interacts with other factors to shape access to belonging for migrants settling in the same host community. To address this gap, we analyze 73 semi-structured interviews with migrants in Utah, USA, to investigate the process and experience of belonging for migrants across permanent, temporary, undocumented, and refugee statuses. While legal immigration status is an important factor shaping (non)belonging, it does not appear to function as a master status for migrant belonging. Rather, we find that legal immigration status works alongside a number of community-level factors—including cultural, social, linguistic, and racial/ethnic factors—to shape belonging for migrants of all immigration statuses. These non-legal, community-level factors emerged as critical features of (non)belonging for many migrants living in Utah. Our findings suggest that, although they cannot change federal immigration policies, local- and state-level governments and organizations can enhance migrants’ access to belonging and wellbeing across many other dimensions.

## 1. Introduction

What does it mean to belong to a community, and why does it matter if someone feels like they belong? The migrant experience can reveal the contours of belonging in a specific society in ways that illuminate the often taken-for-granted formal and informal social structures of everyday life. Belonging also plays an important role in shaping migrants’ (and non-migrants’) health and wellbeing [1,2]. Researchers examining immigrant and refugee experiences of both marginalization and inclusion suggest that non-belonging—and social and structural exclusion—negatively affects migrants’ health and wellbeing [3,4,5,6,7]. They also find the reverse to be true: that “belonging can be utilized and drawn on as a forceful means and resource of social resilience” for migrants, restoring and sustaining their health and wellbeing [2] (p. 2) [7,8]. As such, identifying and improving the factors shaping migrants’ sense of belonging can produce improved social, emotional, and physical health and wellbeing for migrants and their communities [5,9].

As a theoretical concept, belonging involves civic, social, and emotional dimensions [10,11]. Migrants navigate this sense of belonging across household, workplace, and community environments, which can vary and change over time [12]. As a result, there is tremendous diversity of migrant experiences documented in the literature, in the United States and abroad [11]. For example, migrants arrive with different cultural and racial backgrounds [13], often confronting negative framing and racial stereotyping [14]. Migrants also arrive with different legal statuses that affect their ability to incorporate [15,16], and this can vary across region and country [17,18].

As important as this literature is for understanding migrant belonging, it is hard to pinpoint how migrants experience belonging across a host of case studies with different contexts and subjects. Our contribution, then, is to compare the experiences of multiple migrant groups *in the same setting*—refugee, permanent, temporary, and undocumented migrants in Utah. Through this analysis, we are able to consider the salience of permanent, temporary, and undocumented migrant classifications in shaping belonging when compared with other key community-level cultural, social, linguistic, and racial/ethnic factors.

To do so, we utilize 73 semi-structured interviews with migrants living in Utah, collected in 2017–2018 (refugees) and 2019–2020 (all other migrants). Utah has received tens of thousands of refugees for resettlement over the past two decades, and its immigrant population has increased more than 350 percent in the past 30 years [19,20]. As a new immigration destination in a politically conservative state whose political leaders have generally proven sympathetic towards refugees and migrants [21], Utah represents a unique political and demographic setting for understanding migrant settlement and belonging. We examine migrant experiences before, during, and after arrival into the US through in-depth interviews and employ a subject-centered approach that allows us to examine migrants’ subjective experience and understanding of belonging [22].

## 2. Literature Review

### 2.1. Sense of Belonging

Belonging can be difficult to define, both conceptually and affectively [23]. Belonging is intersectional [23,24] and can take many forms, such as national and affective citizenship [25,26], cultural identity as a form of positioning [27], migrant acculturation [28], and place-belongingness [29,30]. We define sense of belonging as migrants’ perception of their ability to incorporate and participate in the host nation [31,32], or how “migrants view themselves in relation to others in society” [33] (p. 948). In essence, a sense of belonging is a psychological feeling in which individuals feel a connection to the people and places where they live [34]. This sense of belonging is dependent on migrants’ experiences and social environment. When migrants face legal, economic, cultural and/or social exclusion, that exclusion negatively influences many of the experiences, opportunities, and outcomes they use to build a sense of belonging [35,36]. Alternatively, inclusion may build a sense of belonging that benefits migrants’ success and wellbeing, while increasing social participation and societal cohesion [37]. The benefits of belonging (or harms of non-belonging) can extend beyond social and economic factors to shape migrants’ physical and emotional health and wellbeing, too [2,38].

Scholars have identified factors that are positively and negatively associated with a sense of belonging [39]. Factors that influence belonging include formal (e.g., legal status) and informal factors (e.g., religious participation) [33]. While navigating life in a new national context, international migrants undergo a range of experiences that affect their sense of belonging, including daily interactions within legal, economic, social, and cultural spheres of society [37,40]. Positive interactions within these different realms of social life can enhance migrants’ sense of belonging, while negative interactions can have the opposite effect [41,42].

Our approach to understanding belonging has an important framing that is often underutilized in the literature: we focus on belonging as defined by the respondents themselves [22,43]. This subject-centered approach allows us to understand the meaning of belonging from migrants’ perspectives and incorporate aspects of belonging derived from interviewees’ experiences, rather than the researchers alone. Interviewee responses highlight five overlapping categories that shape belonging—legal, cultural, social, linguistic, and racial. In the following sections, we briefly review each factor in turn.

### 2.2. Legal Status

US immigration and border policies, combined with migrant legal status, directly affect sense of belonging and opportunities to incorporate [15,16]. US immigration policies place a large share of the migrant population outside of the law, depriving millions of individuals and families residing within the United States of social, economic, and civil rights [44]. Legal punishments directed toward some migrants combined with the rising inaccessibility of citizenship and its associated rights are “playing an increased role in patterns of exclusion” for both migrants and their family members [45] (p. 117) [36,46]. US immigration laws regulating migrant statuses include long-term legal statuses granting a right to work, receive education, and reside within the US, as well as temporary and undocumented statuses which restrict or offer no legal right to work or reside within the US. Policies also create a third category of *liminal* inclusion, wherein individuals (such as students or workers) have temporary or circumscribed rights to live, study, and/or work in the United States for the duration of their visas, without a path to permanent residence or citizenship [47]. (“Liminal” status is a term used by migration scholars to describe statuses that are temporary and in-between permanent status and no status, such student, tourist, and temporary work visas, as well as Temporary Protected Status and DACA [47].) Current immigration laws require migrants to prove self-sufficiency while barring or precluding many individuals from access to the education and work necessary to sustain themselves [48]. Most (would-be) migrants have no access to permanent (or even temporary) legal migration status in the US. When individuals are able to achieve a legal status of permanent residency or citizenship, they gain access to key institutions and greater opportunities for full incorporation in society [36].

Individuals with liminal or no legal status face additional risks in their ability to incorporate and, therefore, in developing a strong sense of belonging [15,47]. Uncertain legal status “permeates many aspects of the immigrants’ lives and delimits their range of action in different spheres, from job market opportunities and housing to family and kinship” [47] (p. 1001). An undocumented status excludes immigrants from the political and economic institutions that allow them to fully participate in and incorporate into society. Without such access to opportunity, migrants, their family members, and the communities in which they live all suffer [49,50]. With restricted or no access to jobs, skills training, or education, among other resources, temporary and undocumented immigrants often struggle to sustain productive and fulfilling lives. Legal status is a structural, concrete factor in the lives of migrants which has lasting effects and consequences but cannot easily be changed. Consequently, scholars have emphasized the dominant role legal status plays in shaping migrant belonging [15,51].

### 2.3. Cultural Familiarity

Migrants in the US come from a wide variety of cultural backgrounds [52], and culture varies between and within migrant groups. Migrant groups do not have one single “set of values, traits, beliefs, and behavioral patterns that are fixed and intrinsic” [53] (p. 180), nor does the receiving society have only one single culture into which migrants may integrate. Rather, both migrant and host groups are multicultural [54]. Migrants from the same country, and even the same city, have diverse cultural backgrounds [55] and bring class-specific experiences that may manifest as divergent cultural practices [13]. It is critical to recognize the heterogeneity of migrants’ backgrounds and experiences, even if they share the same national origin, language, religion and/or other characteristics.

Migrants’ cultural backgrounds and familiarity with host country culture(s) can provide them with cultural capital upon arrival. Cultural capital includes knowledge and skills that promote participation in the host country. Relevant cultural capital can facilitate migrants’ cultural inclusion, while a lack of cultural capital relative to American society can contribute to cultural exclusion. For example, for migrants with an insecure legal status, places of worship can become central locations for establishing social connection and a sense of belonging [56]. Cultural capital can enable migrants to enter into and participate in the host society with a degree of familiarity and comfort [57]. Often, though, broader cultural practices, preferences, and pressures within the host country determine migrants’ cultural inclusion or cultural exclusion, regardless of migrants’ individual efforts to belong [52,57,58,59].

### 2.4. Social Capital

While migrants participate in US culture, they also establish, build, and maintain relationships that allow them to assemble a wider social network. A social network is made up of the interpersonal ties linking migrants and their families, friends, and community members [60]; these relationships provide social capital, which includes resources, information, and access to opportunities. Social networks are dynamic and shift over time [61]. Pre-migration social relationships and networks at the point of destination often motivate migration intentions and decisions and can ease the costs and challenges of migration and settlement [62]. During the initial post-migration period, relationships with those of similar ethnic backgrounds can be particularly valuable [59,61].

Over time, relationships with people of varied social locations can foster increased opportunities, though relationships that transcend ethnic, national, or socio-economic differences may take more effort to establish and may be lacking in closeness or intimacy [61]. Diverse forms of social relationships including brief encounters, acquaintances in places such as churches or workplaces, and friendships play an important role in generating both resource access and a sense of belonging post-migration [56]. Close friendships, in particular, create a sense of being socially embedded or rooted in the new environment. Establishing this sense of belonging is often more difficult for undocumented migrants or asylum seekers with insecure housing and legal status [56].

### 2.5. Linguistic Belonging

Communication abilities influence cultural and social belonging for migrants while also shaping economic opportunities. Language ability is shaped by multiple factors including pre-migration schooling, intentions to settle, age, health, and post-migration resources and services [63,64]. Language often functions as a “symbolic boundary” in negotiations of migrant belonging [65]. Linguistic familiarity, fluency, and accent in the host-country language can shape migrants’ access to belonging by influencing their ability to build friendships, gain work, advocate for themselves and their rights, and identify with the host country [10,65,66]. Among Syrian refugees in the US, shared language ability enhanced belonging through fostering understanding within friendships and inclusion within employment contexts [10]. Other research has identified the isolation and lack of social capital that can occur when US migrant households lack English proficiency [67]. For many migrants, the inability to speak English has been a barrier for accessing information, health care, social services, and/or respect. These barriers were compounded in communities with limited social and economic resources [67].

### 2.6. Racial Belonging

Racism and the racialization of migrants can also directly influence opportunities for belonging [11]. Race has long influenced immigration law [68,69,70], and the history of US immigration law includes many examples of racist and exclusionary policies [58,71] that directly limited some migrants’ ability to settle in the US and indirectly contributed to specific racialized and classed understandings of what it means to be “American” [72]. Belonging in the United States, therefore, is affected by a legal legacy that contributed to racialized views and policies that favor people with particular nationalities, ethnicities, and skin tones.

Race and racism shape life opportunities in the US, including opportunities for migrant integration and belonging [73]. US immigration law has contributed to the racialization of migrants who are not white or middle class [74,75], creating conditions in which many non-white and lower-class migrants are regarded as undesirable and unwelcome [14,76]. Racism and racial exclusion limit belonging, where racialization becomes a mechanism through which migrants are stigmatized and regarded as out-group [14]. The racial stereotypification of migrants “effectively sabotage[es] the possibility of creating a community” and inhibits migrants from enjoying full inclusion [77] (p. 116). Racial and ethnic diversity, or lack thereof, seems to matter most at the neighborhood-level in shaping migrants’ sense of inclusion or exclusion [78]. In studies of belonging, scholars have found that (non)belonging is often shaped by the intersection of racial status with other identities, such as class and religious affiliation [79,80].

### 2.7. Pathways of Belonging

Across these different dimensions, the literature suggests that community-level characteristics—not just regional or national factors—play an important role in immigrant and refugee experiences of (non)belonging [23,59,78]. With these overlapping, community-level factors in mind, this study examines how multiple societal conditions shape migrants’ sense of belonging in the same reception setting. We build on the existing literature by considering how the experiences of migrants with different migration statuses living in the same host community may experience (non)belonging differently across legal, cultural, social, linguistic, and racial planes. We document how these societal conditions positively (or negatively) affect each migrant’s sense of belonging and find that, while legal migration status does play a clear role in shaping belonging, migrants of all migration statuses can find belonging (or feel excluded). To preview our results, we find that multiple factors affect migrant belonging and that there are multiple pathways for migrants to achieve belonging. These findings expand our understanding of the interplay between migration status, cultural and social inclusion, and sense of belonging, as well as the role of community in shaping belonging for migrants with varied immigration statuses. Our findings also suggest the need to expand policy and practical reform efforts beyond legal migration status to include social, cultural, linguistic, and anti-racist interventions that increase migrants’ opportunities for and sense of belonging at the community, regional, and national levels. Such efforts could positively shape health and wellbeing for migrants in the short and long terms.

## 3. Data and Methods

### 3.1. Research Setting

The state of Utah, USA, provides a unique case to understand migrant belonging in a new destination setting. Utah is an emerging migration destination to which thousands of migrants with a variety of statuses are arriving every year [19,81,82,83]. Over the past three decades, Utah’s foreign-born population has grown by more than 350 percent to compose 8.6 percent of the total state population, including tens of thousands of refugees who have resettled in the state [19,20,84]. Utah is the fastest-growing state in the US; its total population increased by over 60 percent between 2010 and 2020—from 2,074,505 to 3,337,975—and its foreign-born population increased by over 75 percent during that same period, from 158,664 in 2010 to 278,336 in 2019 [19,84]. Utah has historically had a dominant monoculture made up of a racially and religiously homogeneous population, but it has rapidly diversified over the past forty years [85,86,87,88]. Foreign-born Utah residents have contributed significantly to Utah’s racial diversification, with most identifying as Latinx (53.9%), Asian (18.2%), 2+ races (22.6%), or “other race” (27.1), and only 16.9% identifying as White only—a stark contrast to the 81.4% of US-born Utah residents who identify as White only [84]. Because increased ethnic diversity is associated with native-born White Americans’ intensified xenophobia, the new and growing diversity in Utah could directly affect immigrants’ integration opportunities and experiences there [89]. Thus far, though, Utah and its political leaders have generally proven sympathetic towards refugees and migrants, despite the state’s large conservative majorities in elected office and the broader population [21]. Collectively, these factors have created a unique political, social, and cultural climate for migrant settlement ideal for the study of migrant incorporation and belonging in new migration destinations in the US [21,86,90].

### 3.2. Sample

This analysis features a subset of interview and questionnaire data drawn from a broader study examining migrant integration across legal migration statuses. The broader study includes qualitative interview and questionnaire data collected from 162 migrants with refugee, permanent, temporary, and undocumented statuses living in Utah, USA. Undergraduate and graduate student researchers and faculty PIs conducted interviews with 88 refugees between June 2017 and November 2018 and with 74 immigrants between April 2019 and November 2020. Project researchers used snowball sampling to identify study participants. To identify potential interviewees, we shared study information and flyers with immigrant- and refugee-serving institutions, on social media, and through word of mouth, listing information about participation criteria and compensation ($20 USD per interview). The criteria for participation were: (1) to have arrived in the US as migrants, (2) be 18 years or older, and (3) have lived in the US for at least 5 years and Utah for at least 2 years at the time of the interview. For this study, we center our analysis on interview and questionnaire data from the 73 interviewees in which sense of belonging was explicitly addressed in the interviews. Given that belonging is central to our analysis here, we limit our analysis to those interviews in which belonging is explicitly discussed. [In the other 89 interviews, belonging was not discussed explicitly during the in-depth interviews.] In order to identify qualifying interviews, we conducted a term search for the word “belonging” in all 162 interview transcripts. All interviews found to include the term in the interview transcription were included in our analysis. From our search, it is clear that this difference in thematic interview content reflects the interviewers’ questions and not any meaningful difference among the study participants. We found no evidence that the interviewees whose responses were not analyzed here have distinctly different understandings or experiences of integration, community closeness, belonging, etc. from those whose responses were included in our analysis. Table 1 summarizes the demographic information of the 73 interviewees—born in 35 different countries—whose responses are included in our analysis.

We examine belonging across four migrant statuses—permanent, temporary, undocumented, and refugee—and study participants were categorized based on their current migration status as described during their interviews. Due to concerns about participants’ safety, only refugee interviewees indicated their legal migration status as arrivals through the U.S. refugee resettlement program. Most interviewees volunteered information about their status during their interviews. For the analysis presented here, we did not include interviews in which a participant’s legal migration status was unclear. (Although this is a limitation of our study, we believe the voluntary nature of disclosing migration status allowed the respondent to reveal the importance of legal status in their sense of belonging.) *Migrants with permanent status* (38 interviewees) are those migrants who possess lawful permanent residency—a status which grants them long-term permission to live, work, and study in the US—or have transitioned from permanent residency to citizenship. A majority of permanent migrants access this status through familial relationships with a US citizen spouse, parent, or sibling [91]. A smaller number access permanent residency through high-skilled employment after working on a temporary work visa for several years. Permanent migration status offers a path to citizenship. *Migrants with temporary status* (13 interviewees) are those migrants who gain entry into the US for a limited period of time. Many migrants fall under this category, including foreign students, temporary workers and their spouses, and tourists. (Some visas, including tourist visas, are considered “non-immigrant” visas. In this analysis, we consider participants who were living in the US at the time of the interview with a valid non-immigrant visa as “temporary” migrants.) Each temporary status includes different restrictions on employment and duration of stay [92,93]. Most temporary migrants do not have a clear path to permanent residency or citizenship. *Migrants with undocumented status* (9 interviewees) are those migrants who overstayed a visa or entered the US without being lawfully processed through a port of entry. These migrants do not have a right to work or reside in the US. An exception is made for those individuals who qualify for DACA (Deferred Action for Childhood Arrivals). They are temporarily protected from deportation and granted the ability to work and study for a limited (but renewable) period of time. All other undocumented migrants face the threat of deportation. Undocumented migrants do not have a path to permanent residency or citizenship; if they leave the US, they are generally subject to years-long bans from the US before being able to return to the US legally on a temporary or permanent basis. *Refugees* (13 interviewees) are internationally displaced persons who are approved for permanent settlement in the United States after they experience war, natural disaster, or persecution based on their race, religion, nationality, political opinion, or membership in a particular social group [94]. After passing several screening and vetting processes, refugees are resettled in a specific state and city in the US. (Almost all refugee interviewees were resettled in Salt Lake City, Utah.) As part of the resettlement process, refugees receive support in accessing resources for housing, education, healthcare, employment, and language training during their initial months post-arrival. Refugee and asylee statuses provide direct paths to lawful permanent residency and citizenship.

### 3.3. Interview Process

The interviews were divided into two parts. During the first portion, interviewees were asked to fill out a brief questionnaire, which asked for basic demographic details and information about the interviewee’s religion, access to resources, wellbeing, and health. During the second portion of the interview, we asked interviewees open-ended questions about their experience as migrants, including: the reasons they decided to migrate to the US, how their realities post-migration met their expectations pre-migration, opportunities and challenges they faced since settling in Utah, what success and integration looks like for migrants, how factors such as class and gender affect their experience, and whether they feel integrated in Utah and the US. The interview structure allowed for follow-up questions and in-depth conversations. Interviews lasted from thirty minutes to a few hours, averaging about an hour in length. Interviews were recorded with consent of the interviewees and then transcribed by members of the research team. The interview recordings, transcriptions, and questionnaire data were entered into secure databases accessible only by the project manager and researchers. We then used NVivo and Dedoose for data management and analysis.

### 3.4. Analysis

In our analysis of the 73 interviews included in this study, we examined interviewees’ discussions of belonging, noting their assessments of their own belonging and the reasons they offered for why they (do not) feel like they belong. Many interviewees explained that their belonging was affected by multiple factors, and they often used the terms belonging and integration interchangeably. We organized their responses regarding belonging into a spreadsheet along with demographic, health, and wellbeing data from the questionnaire. In accordance with interviewees’ discussion of their understandings of and sense of belonging, we categorized each participant’s belonging (labeled as yes, no, or sometimes) on the spreadsheet and the reasoning they gave for why they feel they (do not) belong. We coded those responses to note which societal factor(s) interviewees cited in their discussions of belonging. We organized findings around the five societal factors interviewees identified as critical to belonging; our analysis of those factors follows below. 

Migrants’ experiences are incredibly complex, and we have only been able to examine a fraction of them here. Belonging is not solely impacted by themes we explore in this study (i.e. migrants’ legal and socio-cultural experiences); other characteristics such as economic opportunities, gender, or family structure may also affect their sense of belonging. Those factors were not included or emphasized here because they did not emerge in the analysis as leading factors shaping belonging among the interviews analyzed.

This study is limited by its sample size. The 73 individuals we included in this study are a small fraction of the 278,000 migrants in Utah and almost 50 million migrants currently living in the US [89,95]. Despite these limitations, we believe that this subject-centered examination of belonging for a diverse group of migrants contributes an important perspective to our understanding of the multiple pathways for migrant belonging and wellbeing. We hope that by “refocus[ing] attention on the migrant, and on migrant–host-society relations, [… we are] making room for multiple spaces of belonging” [11] (p. 1596)—a critical call in the literature.

## 4. Findings

**Figure 1:**
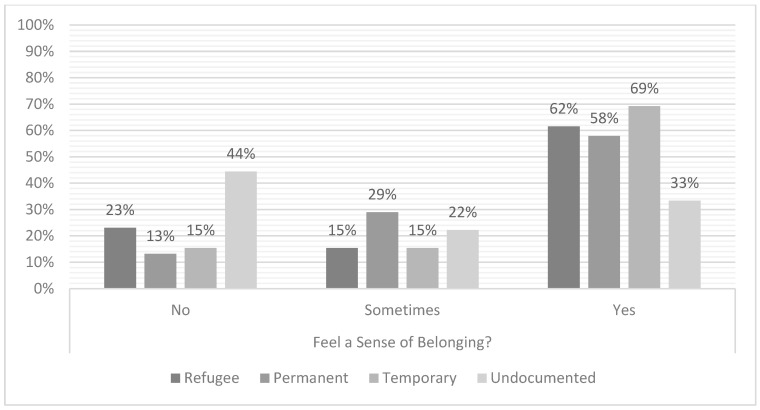
Sense of Belonging by Legal Status within Categories.

Among the 73 participants whose interviews were included in this analysis, 14 interviewees (19% of the sample) expressed feeling that they do not belong; 17 interviewees (23%) expressed feeling like they sometimes belong; and 42 interviewees (58%) expressed feeling that they belong in the United States and/or Utah. Migrants with permanent, undocumented, refugee, and temporary status were represented in each of the categories of belonging. (See Table 2 and Figure 1; for additional demographic information by sense of belonging, see Appendix A.)

As shown in Table 2 and Figure 1, the majority of interviewees, regardless of their legal migration status, expressed feeling like they belong; but more than 40 percent of interviewees across migrant statuses expressed feeling that they belong only sometimes or never, including migrants with long-term legal authorization to live and work in the US. These findings alone suggest that legal migration status is not the sole determinant of migrant belonging, though legal status clearly plays a role in shaping belonging (especially for undocumented migrants). Across all interviews, five societal factors—legal, cultural, social, linguistic, and racial—emerged as central components in interviewees’ understandings and experiences of (not) belonging. Collectively, their responses suggest that several factors contribute to belonging, providing multiple pathways (and, for some, barriers) to belonging.

### 4.1. Legal Belonging

As shown in Table 2 and Figure 1 above, migrants’ legal status did not unilaterally determine belonging, but interviewee responses suggest that legal migration status still plays a direct role in shaping migrants’ access to opportunity and sense of belonging. Migrants without permanent legal status confront steep barriers to opportunity, explaining that they are not able to succeed in the “the country of opportunity” (Gabrielle, undocumented status), even though, for most migrants, “all they want is a job and an opportunity” (Diego, formerly temporary, now permanent status). David (undocumented status) described how his mother’s undocumented status denied her the “opportunity to create a different life for herself.” As Rodrigo (undocumented status) explained, migrants could study, work, and live more freely if they did not have the “handicap” of being an undocumented migrant. Instead, many migrants feel held back by a lack of permanent legal status. Their status does not “let them do what [they] want” (Juan, undocumented status), gives them a feeling they have “no power to do anything,” that they are “hindered,” “can’t reach [their] full capacity,” and that their “foot is tied down” (Olivia, temporary status).

Alfonso (formerly temporary, now permanent status) shared how his life changed after gaining permanent residency: “I have a better job. I can work off campus. I’ve got a green card. I realize now I can figure out life more. I have more opportunities.” But many migrants who secured permanent migration status spent significant time and resources to achieve that permanent status. Several of those interviewed described arduous immigration processes. When the process started, many were not aware “how much money it would take, how long it would take” (Gabrielle, undocumented status). Others shared that they achieved legal status after a “long time” (Carla, formerly temporary, now permanent status; and Jose, temporary status) and a “long process” that was “expensive” (Pedro, formerly undocumented). Some were even separated from their families for months (Pedro, formerly undocumented) or years (Valeria, formerly undocumented) as they went through the immigration process to adjust to a permanent legal status. These inefficiencies are due to a complex immigration system that requires migrants with a pathway to permanent status to invest massive amounts of time, resources, and effort towards gaining it.

Acquiring permanent resident status allowed many interviewees to feel a sense of belonging. In addition to helping migrants see that they now “belong” in the United States (Mandy, previously undocumented), permanent residence was recognized as a step towards “becoming a [citizen] of a great, big country” (Ali, refugee). The importance of permanent status for belonging can be seen in Sebastian’s description of the elusive nature of belonging for him as an undocumented immigrant:

*I’ve always had to go above and beyond to prove that I belong. One day I want to actually be a citizen, you know? [starts crying] And I, and I think that that will probably be the most important day of my life, you know? Cause it’s a chance to finally say like, “Look, I really do belong.” I could live anywhere here and know that this was my home because I’ve worked so hard to belong here.*

Knowing one has permanent status within the US signals belonging to migrants and non-migrants alike, acting as official evidence of membership and, thus, belonging.

Permanent resident status also provides specific legal rights. Naomi (temporary status) said she would be able to feel like she had a place if she had “similar rights [as] people from here” and this would allow her and others to say, “this is your country, and that, you know, you’re from the United States.” Wangmo (refugee), recounted how citizenship gave them “somewhere to belong”, and Sonam (refugee) expressed that citizenship gave her “some place to call home.”

But as Table 2 and Figure 1 also show, legal status is not the sole determinant of belonging for migrants. Not only did many interviewees with permanent status feel they belong only sometimes or never, many interviewees without permanent migration status found belonging beyond legal inclusion. Cultural, social, linguistic, and racial factors, in addition to (or in spite of) legal migration status, provided other pathways to belonging for interviewees.

### 4.2. Cultural Belonging

Cultural practices and connections played a profound role in shaping belonging for many interviewees. (Culture is a difficult concept to define, as any one geographic region contains many different cultures and understandings of culture. In our analysis, we use interviewees’ own understandings and descriptions of culture.) Participants in our sample had a number of explanations for finding cultural belonging. Perhaps because culture is complex (and hard to explain) and each migrant has a unique culture (composed of multiple elements), interviewees spent the most time explaining how elements of culture, and their familiarity with those elements, shaped their sense of belonging. Migrants specifically described finding belonging through cultural familiarity with and embracing elements of national and local culture, religious participation (especially in the predominant religion of the local context), and engaging with co-ethnic communities.

Some interviewees described how they see taking part in the general “American culture” as a signifier of integration. Sebastian, an undocumented immigrant who came to the US as a child, explained why, despite his lack of citizenship, he thinks of himself as American:

*I feel like I’m an American. Yeah. Right. But like the arbitrary designation of citizen is… I’ve never even felt like remotely close to that, you know? Right. But I definitely feel American, you know, like I grew up in the same high schools. I watched the same movies growing up in the same language. I played the same video games. I watched the same TV shows every Saturday morning. I read the same comic books and, like, I think I went to the same goddamn church. I feel like there is nothing that separates me from my American friends, you know?*

Sebastian has been participating in what he regards as the “typical” American life since he was little. Because he is doing things that those around him are doing, he does not see himself as different. Rather, he sees himself as belonging culturally, even if he does not belong legally.

Other participants such as Andres (temporary status) described belonging in cultural terms: “being able to speak English, […] enjoying the food because everybody eats, […] sporting events, […] just like going and being part of the crowd.” Irene (permanent status) also regards herself as belonging because “in my house, the language spoken is English, the food that is eaten is typical food from here, the things we do are typical American.” In these ways, having cultural proximity to or participating in the dominant culture can shape migrants’ integration and sense of belonging, regardless of their legal migration status.

Not all migrants who find cultural belonging completely immerse themselves in the host culture, seeking instead to strike a balance between their home and host cultures. Many of these migrants find their multicultural approach as a pathway to belonging. Alfonso (permanent status) “took some aspects of their [US] culture, but the core is still me;” similarly, William (permanent status) believes that “for integration, I do not think it is losing your cultural heritage but it’s coming into America and taking what you think is best and adopting those things.” Cristina (permanent status) expressed a desire to not only merge cultures in her own life, but also in the lives of her children: “I want to keep my culture and teach my kids my culture. [...] I want them to learn the culture of the United States, but I really want to keep what makes me feel that I am here, but I am from there.” Yu Yan (temporary status) shared similar sentiments when she explained that it is important:

*To have the ability to build your own culture. Shape the person you want to become. Also hold onto those values you have for yourself. As immigrants we come with our own culture and background. You are constantly evolving and changing. Every day you are immersing yourself with so many different cultures that may be different or hard.*

Like Yu Yan, some migrants see the importance of understanding and participating in their host country’s culture while also maintaining their home country’s culture as a way to facilitate a sense of belonging.

Other migrants shared that their sense of belonging was tied to co-ethnic community membership and activity. These migrants were able to find a big enough part of their “home” culture in the US to feel like they belong here. Noelia (permanent status) was able to preserve her identity within the greater community by intentionally seeking out community among the Mexican population in Utah:

*When we moved back from Mexico a few years ago, we started going to our English “ward,” our church congregation that was English-speaking. But I really wanted to be in a Spanish ward. And a couple of years after, we moved to the Spanish ward because that’s where my husband was assigned to work and serve, and I felt right at home there. So, at church, surrounded by Hispanics, or Spanish speakers, is one of those places where I love it. But also, when I surround myself with women who can also relate to my heritage, whether it’s because they speak Spanish or because they too have Hispanic heritage, that too has made me feel right at home. And I hadn’t realized that was one of the things I needed. Like, I have a ton of friends who are English speakers, and I love being with them, but I hadn’t realized how much—how I am my complete self when I can be Spanish Noelia.*

This completeness of self that Noelia described is something other interviewees sought by finding ways to preserve their identities. Some recounted their efforts to maintain a smaller community within their new host community, like Carla (permanent status), who explained that “in our case, in my house so to speak, we live like [we’re] in Ecuador. We cook like we’re in Ecuador, we always speak Spanish, we try to have decor that reminds us of our country. So, we conserve our own values.” By finding ties to their home country’s social and cultural networks, some migrants are able to maintain a sense of belonging even within larger and mainstream US culture, like Beth (permanent status), who says of the Filipino community in Utah that “every time I’m with them, it feels like home.”

Another aspect of cultural belonging for some migrants in Utah stems specifically from the state’s unique religious context. Approximately three-fifths of the interviewees were members of the Church of Jesus Christ of Latter-day Saints (LDS Church), the majority religion in Utah whose leaders founded the state and to which more than half of Utah’s population claims affiliation [86]. (Some of the undocumented, temporary, and permanent migrant interviewees were members of the LDS Church prior to migrating to the US; none of the refugee interviewees were LDS before arriving to Utah. Some interviewees, including refugees, joined the LDS church after settling in Utah.) LDS interviewees, particularly those who were members of the faith prior to migrating to Utah, discussed how their familiarity with the LDS Church, including knowledge of the values and actions typical of an LDS Church member, helped them to quickly feel a sense of affinity and belonging in Utah. Being familiar with and subscribing to LDS culture, as well as having others understand why she participated in that religious community (something she did not experience prior to migrating), helped Beth (permanent status) feel like she belongs in Utah. She shared: “When I got here, everyone was LDS. Here everyone just knows [about your faith] and you don’t have to explain yourself. I think it’s cool because I like my people.” Beth sees LDS Church members as “her” people. Other interviewees also described how their membership in this LDS community that crosses international borders contributed to their sense of belonging in Utah. Amy (permanent status) shared: “We have Sunday church meetings and [activities for women and youth], that’s why we always feel like we belong to this area. Because we attend the same [congregation as our neighbors] and then it makes me feel like there’s no big differences.” Participating religiously allowed migrants to highlight the similarities they held with those around them, rather than highlight their differences.

Those who were LDS before migrating to the US already participated in the LDS Church and were familiar with church norms and culture before moving to Utah. Rather than struggling to adapt, these individuals were able to quickly participate in a community that was familiar to them and in which they had already participated and found meaning. Being familiar with the traditions and practices within the LDS Church helped LDS migrants adapt and be part of Utah culture, even when their national cultures may be quite different from broader American culture. Emilia (permanent status), for example, was not familiar with “all of US culture, exactly, but Utah culture” and she “got immersed into the BYU culture, which was even more kind of in a bubble, restricted and different. I liked it though. I mean that I felt comfortable in that kind of environment.” (BYU [Brigham Young University] is a university sponsored by the LDS Church located in Provo, Utah, about 40 miles south of LDS Church headquarters in Salt Lake City.) Eva (temporary status), before moving to the US, knew “BYU and BYU Idaho were religious schools, based on the values we have at church. The new lifestyle didn’t impact me the way I thought it would. It was not a huge change.” Knowing the LDS culture allowed Emilia and Eva to comfortably participate in this predominant Utah subculture as soon as they arrived in Utah.

Not all migrants had this religious familiarity upon arrival or experienced religious belonging in Utah. Many participants joined the religion after settling in Utah, and most of the refugee participants had not heard of the LDS Church prior to migrating to Utah. For migrants unfamiliar with the LDS Church, confronting this unique Utah subculture leaves some feeling like “double” (national and religious) outsiders [87,96]. The predominant and insular LDS culture in Utah led to an ongoing combination of pressure to convert and explicit exclusion from many social and cultural gatherings for non-LDS migrants. An (permanent status) explained that it has been “hard to find the right friends because everybody I talk to, they try to convert me.” She and other interviewees discussed how declining those overtures to conversion often resulted in their exclusion from formal and informal social events (including non-religious activities). Additionally, some participants who were part of the LDS Church prior to migrating felt a disconnect within LDS Church settings in Utah, complicating cultural familiarity and affinity as a pathway to belonging. Desi (permanent status) and Dayana (permanent status), who were both active in their local congregations of the LDS Church before immigrating to Utah, withdrew from religious participation in the LDS Church in Utah after encountering the “judgmental” (Desi) culture in which people were always “criticizing” (Dayana) you. Rather than finding a “home away from home” through their continued religious activity, some LDS migrants in Utah encounter a hostile, superficial, and judgmental “bubble” (Emmanuel, permanent status) at church and among co-religionists that leads them to feel a lack of belonging within a religious culture that had been a primary source of identity and belonging pre-migration.

### 4.3. Social Belonging

Relationships and social networks facilitate belonging by providing encouragement for migration, giving migrants access to resources upon arrival, acting as “evidence” of belonging, and giving migrants a community to which they can belong. Many migrants establish social networks with US-based family and friends before they migrate to the United States. A number of interviewees had relatives who already lived somewhere in the US. Some had not “seen [their] sister in a long time” (Beth, permanent status), “were separated for three years from [their] dad” (Valeria, permanent status), “already had family here” (Carla, permanent status), had “parents [who] came first” (Laura, permanent status), or “had some family in town” (Lucas, undocumented status). Other respondents, particularly those affiliated with the LDS Church, had friends through international (religious) social networks who inspired or helped them to come to the US, and often to Utah specifically. For these interviewees, their international social networks motivated and even facilitated their international migration, even when the interviewee did not have clear legal or economic avenues that would help them migrate successfully.

Upon and after arrival, migrants’ US-based friends and family can help them find needed resources. Alejandra (permanent status), for example, attributes her success at work to her relationships: “In regard to working, it has all been thanks for networking with friends from school. I have kept that contact with people in my field. I have had opportunities.” Friends may provide short- or long-term opportunities that allow migrants to participate in society. Families also often provide the resources and support migrants need. Eva (temporary status) said, “I think it has a lot to do with family—they are the ones I call when I need help.” Other acquaintances and connections offer the support needed as migrants arrive, helping migrants “fit in” (Nicolas, undocumented status), experience “growth” (Gabrielle, undocumented status), “communicate” and “improve” (Desi-permanent status), and find an “instant connection” (Andres, temporary status).

Often relationships themselves are what help a migrant feel like they belong, as many interviewees described having friends and other relationships as evidence of belonging. Being able to make friends was seen by many interviewees as a rite of passage. Everyone else around them had a social network (it seemed “normal” to have one) and once they had one of their own, it meant they also belonged. For Andres (temporary status), having American friends and living a daily life like theirs is evidence of his belonging: “I have friends. I go to school, and I just live a normal life, like any other American-born teenager.” Charlotte (temporary status) feels similarly, observing: “I’m just here having fun, making friends...why wouldn’t I belong here?” For Lexi (permanent status), this belonging can happen even without seeking it: “I think that when you are working and going to school somewhere, even if you don’t want to integrate, like those things integrate you because you go to school, you learn […]. You’re spending time with those people and that, it just starts to rub off on you.” As migrants are able to fulfill social expectations of mainstream US society, like making friends, they increasingly feel a part of US society. Overall, having social networks is an important facilitator of belonging because having friends allows migrants to find similarities with others around them, rather than emphasize their differences.

Some migrants, however, do not have strong social networks and, for many of them, their lack of social connections and interactions can hinder their sense of belonging. Without strong relationships, many migrants do not have full access to their communities or the resources within them. Furthermore, their lack of connection to the broader community may lead them to feel like they do not belong. Abdul (permanent status) believes that belonging is related to how strong of a connection you have with the community. He reflected on the importance for him and other migrants to “gain inroads into the local community or some of the community or some of the person[s].” Acknowledging that he is not “a gregarious person,” Abdul explained that he struggled to build connections that helped him feel like he belonged. When not able to have social interactions with others, migrants may feel a lack of connection to the community. As Isaro (refugee) described: “It’s kind of my fault I didn’t get involved when I was [in college] but, I don’t know. It was just hard to like to connect. And I don’t know, maybe I feel like, ‘cause, well, like I’m different…Before I join anything, I have to find this little connection.” For many interviewees, building strong social relationships is key to creating a sense of belonging, even if they, like Lusamba (refugee), do not feel it yet: “Since I came here, I didn’t belong. I want to be friendly with different people so I can learn from them, [and] they can learn from me. That’s helping me to adjust”.

### 4.4. Linguistic Belonging

Many interviewees identified mastery of the English language and the ability to communicate with others as another important facilitator of belonging. Being able to communicate well with others helped many confident English-speaking interviewees feel like they belong. Li Jun (temporary status) had a familiarity with English before migrating because he had attended American schools abroad. Given this linguistic fluency, Li Jun concluded that, “for the most part, I could integrate easily because there was no language barrier”. Sam (permanent status) shared similar thoughts about the need for mastery of the English language. He said, “I think at the basic level, integration is getting to the point where you can function in a society. And that may mean different things. I think learning the language is a big part of that. I think it’s hard to integrate fully if you don’t know the language because you’ll always be at a disadvantage if you can’t do that”.

Migrants who have not yet mastered the language often feel a sense of alienation. Raul (permanent status) shared, “If someone hears me doing something in Spanish, they try to intimidate me and question what I came to do in this country and tell me I must speak English like them. I tell them I’m learning; to be patient”. Phil (permanent status) also felt a sense of pressure to learn English, when he said: “I feel like moving here and understanding like, I should probably speak English, you know, people expect me to speak English.” Dayana (permanent status) recognized that her lack of ability to communicate was “the most challenging” aspect of trying to fulfill her goals in Utah, and it made her feel like she did not have a voice. This kind of alienation experienced by those who did not fully master the English language hindered their belonging, even when they held a permanent legal status.

### 4.5. Racial Belonging

Interviewees often explicitly mentioned race when speaking of their belonging (or lack thereof), with regard to both race-based discrimination and a lack of diversity in many Utah communities. For interviewees perceived as non-white, their race and ethnicity were noticed by those around them and has been used by others as a mechanism to exclude them in formal and informal interactions. When Diego (permanent status) passed through customs at the airport, he was asked whether he had enough money to travel, and when answering that his family owned a hotel, the officer asked him whether it was a “two-room hotel full of cockroaches.” Mandy (permanent status) noted that when she would go to “other people’s houses and they’ve never seen, like, a Hispanic in their home or something and it might have made me feel like they were kind of scared that I was just going to do something”. Similarly, Alfonso (permanent status) went on a hike with friends and noticed there “was this white guy who was so annoyed I was there, and I didn’t know why. Come to realize he didn’t really like Hispanics that much.” Carolina (permanent status) has been told to “go back” to her country of birth, even after being in the US for over two decades. For many interviewees, these racially exclusionary experiences (which were not isolated events) made them feel like they did not, or could not, belong.

A lack of racial diversity in Utah also affected the way that migrants saw their belonging. When Diego (permanent status) first arrived at his Utah elementary school, he realized he “was the only brown kid. I was the only colored kid in the whole school.” Li Jun (temporary status) noted that there “aren’t many Asians in Utah and it was easy to be singled out as the only Asian in class.” Interviewees with lighter skin or hailing from countries that are considered “whiter” experienced racial inclusion, like Alejandra (permanent status), who noted that “because I am fair, my skin is light, I have not experienced that same discrimination [as my] peers from the same country.” Georgi (permanent status) expressed a similar sentiment, describing that, in his interactions with US-born Americans “everybody was nice to me. Sadly, I think it’s because I was an immigrant from Belgium. It would have been different if I was from Mexico or Haiti.” Migrants who can fit into the racial or ethnic majority expressed they had an easier path to finding a sense of belonging in the US because their race and ethnicity did not single them out as being different. (For more discussion of how racism shapes integration and belonging for immigrants in Utah, see [87].)

## 5. Conclusions

We embarked on this study to understand the ways in which migrants across legal migration statuses find belonging in a similar receiving context. As important as legal status can be for belonging, we found surprising pathways beyond legal migration status through which interviewees derived (not) belonging. Our sample is composed of a diverse group of migrants: they vary in age, gender, birth country, religion, and length of stay in the US. They also vary in their legal status: some are students or workers, some are permanent residents or citizens, some are refugees, and some have no legal status. Despite the well-documented role of legal status shaping migrant belonging, our sample showed great variety in belonging across legal statuses (see Table 2 and Figure 1). While we find that legal belonging can influence a broader sense of belonging among migrants, it is not the sole determinant of, or pathway to, belonging. There is more than one factor that affects belonging, and there is more than one way to achieve belonging. Cultural, social, linguistic, and racial factors all offered additional avenues (or barriers) to belonging.

Theoretically, our analysis makes two important contributions to the literature on migrant belonging. First, we include migrants with a variety of legal immigration statuses in our analysis. Migrants receive very different levels of official legal, economic, cultural and social support—support that could directly contribute to opportunities for and sense of belonging—depending on their migrant status category when they arrive to the United States. Yet we find that migrants across all four migration categories find (and do not find) belonging, including refugees (who undergo a lengthy vetting process prior to resettling and receive comprehensive support and a pathway to citizenship upon arrival to the US) and undocumented immigrants (who receive no support and are barred from legal work and residence in the US). We find that, while permanent legal migration status does serve as a pathway for belonging for some migrants with those statuses, it does not guarantee belonging. Similarly, while lack of permanent legal migration status is a barrier to belonging for some migrants, many find pathways to belonging without permanent status. Our inclusion of migrants with multiple and varied migration statuses reveals the nuanced role of legal migration status in contributing to migrants’ opportunities for and sense of belonging.

Second, by grounding our analysis in subject-centered definitions of belonging, we have been able to capture unquantifiable contributors to belonging that researcher-centered definitions often overlook. As described by William (permanent status) in this manuscript’s title, interviewees found belonging when “connecting [with] their communit[ies]”—and connecting with their communities happens in many ways and across many different dimensions. Participants’ responses emphasize the importance of local context in shaping immigrant experiences of belonging, perhaps especially within new immigration destinations. As a result, we encourage scholars and policymakers to see migration, integration, and belonging as largely community-based phenomena, even if they are influenced by policy set at the national level. Local- and national-level policies, practices, cultures, and social norms collectively shape opportunities for and barriers to belonging, opening up multiple pathways for migrants to find and experience belonging in their daily lives.

We offer a new conceptual lens, *community-conditioned belonging*, to emphasize the layered pathways to and conditions that support belonging and wellbeing for international migrants. Community-conditioned belonging highlights that community resources, cultural mores, opportunities to build relationships within the community, and local (anti)racism all shape the place-specific quest for belonging, in addition to—or in spite of—legal migration status and other conditions determined at the national level. This also means that community-level conditions that inhibit belonging can overpower permanent legal status and other national-level, “official” markers of belonging. Community-conditioned belonging suggests that communities play a direct role in migrants’ (non)belonging and can actively influence migrants’ opportunities for and experiences of belonging [23,59,78]. Communities have an interest in supporting migrants’ sense of belonging, as belonging directly influences key measures of health and wellbeing [2,87].

While our interviewees’ descriptions of (not) finding belonging acknowledged the role of social and legal structures as gatekeepers to belonging, their responses also demonstrated how agency, experience, and opportunity also shaped access to pathways of belonging. These findings suggest that localized and individualized efforts to increase migrant belonging can succeed, in addition to ongoing advocacy for needed changes in national-level policies and programs. Consequently, this study has implications for local, state, and federal policies that promote opportunities for migrant belonging. First, migrants should have a wider variety of paths to permanent residency and citizenship. With more flexible (and efficient) paths to legalization, more migrants (including students and workers) will be able to contribute to their host society and achieve meaningful social, economic, and cultural inclusion. Second, work authorization and protection from deportation should be more accessible for temporary and undocumented migrants. This will allow migrants to more fully contribute to the local, state, and federal economy. Third, we find that cultural familiarity and co-ethnic community connections can create one pathway to belonging for migrants. Local and state governments and community-serving organizations may be particularly effective in innovating policies to aid cultural inclusion for migrants. We also need expanded opportunities for language training, outreach and services to underserved communities, and support for organizations that increase cultural understanding. Local and state governments and organizations can also foster social connections between migrant and non-migrant communities. Not only do migrants need cross-cultural relationships to increase belonging, but both migrants and non-migrants likely benefit from the resources and opportunities that come from such networks. Any of these policy suggestions will aid and encourage belonging for migrants from all legal, economic, social, and cultural backgrounds. Despite barriers to inclusion, policy makers and practitioners can continue to encourage and implement such efforts necessary to help migrants achieve belonging.

## Figures and Tables

**Table 1:** Summary of Interviewee Demographic Information by Migration Status.

Demographic Category	Total Study Pop.		Permanent		Refugee		Temporary		Undocumented	
	n	%	n	%	n	%	n	%	n	%
Total	73	100	38	52	13	18	13	18	9	12
Sex										
Male	33	45	15	39	6	46	5	38	7	78
Female	40	55	23	61	7	54	8	62	2	22
Education Level										
At Least Some College	63	86	35	92	11	85	13	100	4	44
High School Diploma	8	11	1	3	2	15	0	0	5	56
Other	2	3	2	5	0	0	0	0	0	0
Age										
20–29	35	48	15	39	3	23	10	77	7	78
30–39	14	19	5	13	5	38	3	23	1	11
40+	24	33	18	47	5	38	0	0	1	11
*Average*	35		39		36		27		28	
Marital Status										
Never Married	24	33	7	18	3	23	7	54	7	78
Married	40	55	25	66	8	62	5	38	2	22
Divorced/Separated	9	12	6	16	2	15	1	8	0	0
Years in US										
5–9	30	41	10	26	7	54	12	92	1	11
10–19	20	27	11	29	4	31	1	8	4	44
20+	23	32	17	45	2	15	0	0	4	44
*Average*	15		19		13		6		18	
Employment Type										
Full Time	38	52	19	50	9	69	7	54	3	33
Part Time	20	27	10	26	2	15	4	31	4	44
Unemployed	5	7	1	3	1	8	2	15	1	11
Other	10	14	8	21	1	8	0	0	1	11
Religious Affiliation										
LDS	45	61	29	76	3	23	8	62	5	56
Other	16	22	3	8	10	77	1	8	1	11
None	7	10	3	8	0	0	3	23	2	22
Not Specified	5	7	2	5	0	0	1	8	1	11
Geographic Region of Birth										
Canada	2	3	2	5	0	0	0	0	0	0
Mexico	11	15	4	11	0	0	2	15	5	56
Central America + Caribbean	6	8	5	13	0	0	1	8	0	0
South America	22	30	16	42	0	0	4	31	2	22
Europe	7	10	4	11	0	0	2	15	1	11
Africa	8	11	1	3	7	54	0	0	0	0
Asia	17	23	6	16	6	46	4	31	1	11

**Table 2:** Sense of Belonging by Legal Status.

	Feel a Sense of Belonging?			
	No	Sometimes	Yes	
Refugee	3	2	8	13
Permanent	5	11	22	38
Temporary	2	2	9	13
Undocumented	4	2	3	9
**Total**	14	17	42	73

## Data Availability

Due to privacy concerns, the data are not publicly available but may be made available upon request to the corresponding author.

## Appendix A

**Table A1:** Summary of Interviewee Demographic Information by Sense of Belonging.

Demographic Category	Total Study Pop.		Belonging = Yes		Belonging = Sometimes		Belonging = No	
	n	%	n	%	n	%	n	%
Total	73	100	42	58	17	23	14	19
Sex								
Male	33	45	19	45	8	47	6	43
Female	40	55	23	55	9	53	8	57
Education Level								
At Least Some College	63	86	38	90	14	82	11	79
High School Diploma	8	11	3	7	2	12	3	21
Other	2	3	1	3	1	6	0	0
Age								
20–29	35	48	19	45	8	47	8	57
30–39	14	19	9	21	3	18	2	14
40+	24	33	14	33	6	35	4	29
*Average*	35		35		34		33	
Marital Status								
Never Married	24	33	11	26	6	35	7	50
Married	40	55	25	60	10	59	5	36
Divorced/Separated	9	12	6	14	1	6	2	14
Migration Status								
Permanent	38	52	22	52	11	65	5	36
Refugee	13	18	8	19	2	12	3	21
Temporary	13	18	9	24	2	12	2	14
Unauthorized	9	12	3	7	2	12	4	29
Years in US								
5–9	30	41	18	43	6	35	6	43
10–19	20	27	11	26	4	24	5	36
20+	23	32	13	31	7	41	3	21
*Average*	15		15		17		14	
Employment Type								
Full Time	38	52	24	57	9	53	5	36
Part Time	20	27	9	21	3	18	8	57
Unemployed	5	7	4	10	1	6	0	0
Other	10	14	5	12	4	24	1	7
Religious Affiliation								
LDS	45	61	24	57	14	82	7	50
Other	16	22	11	26	2	12	3	21
None	7	10	3	7	1	6	3	21
Not Specified	5	7	4	10	0	0	1	7
Geographic Region of Birth								
Canada	2	3	2	5	0	0	0	0
Mexico	11	15	7	17	3	18	1	7
Central America + Caribbean	6	8	1	3	4	24	1	7
South America	22	30	14	33	5	29	3	21
Europe	7	10	3	7	1	6	3	21
Africa	8	11	3	7	2	12	3	21
Asia	17	23	12	29	2	12	3	21
